# Phenytoin Toxicity During Neoadjuvant Concurrent Capecitabine and Radiation Therapy for Rectal Adenocarcinoma: Case Report of a Drug Interaction

**DOI:** 10.7759/cureus.3625

**Published:** 2018-11-23

**Authors:** Clare McGrath, Amy Munro, Rakesh Goel, Kelly Linden, Kristopher Dennis

**Affiliations:** 1 Radiation Oncology, The Ottawa Hospital Cancer Centre, Ottawa, CAN; 2 Radiation Oncology, McMaster University, Hamilton, CAN; 3 Oncology, The Ottawa Hospital Cancer Centre, Ottawa, CAN; 4 Radiation Oncology, The University of Ottawa, Ottawa, CAN

**Keywords:** capecitabine, drug interaction, phenytoin, rectal cancer, 5-fluorouracil

## Abstract

Phenytoin toxicity occurs when serum levels exceed the therapeutic level, leading to symptoms such as nystagmus, slurred speech, and decreased coordination. This toxicity is sometimes caused by drug interactions. Interactions between phenytoin and capecitabine are not commonly documented. We report the case of a 52-year-old man taking phenytoin for atypical meningioma who developed symptoms of phenytoin toxicity while receiving capecitabine in the treatment of rectal adenocarcinoma.

## Introduction

Phenytoin is an anticonvulsant drug commonly prescribed for the prevention and treatment of seizures. When a patient’s serum levels of phenytoin exceed the therapeutic level, phenytoin toxicity can occur with symptoms such as nystagmus, ataxia, slurred speech, decreased coordination, and confusion. Phenytoin toxicity can be caused by overdose, dose adjustments, alterations in physiology, or drug interactions [1].

Publications describing the interactions of phenytoin with chemotherapeutic agents are limited [2], but a few report toxicity due to a presumed interaction with the commonly used capecitabine. Herein, we report the case of a 52-year old man taking phenytoin for atypical meningioma, who developed dangerously high serum phenytoin levels and toxicity while receiving capecitabine in the treatment of rectal adenocarcinoma.

## Case presentation

**Figure 1:**
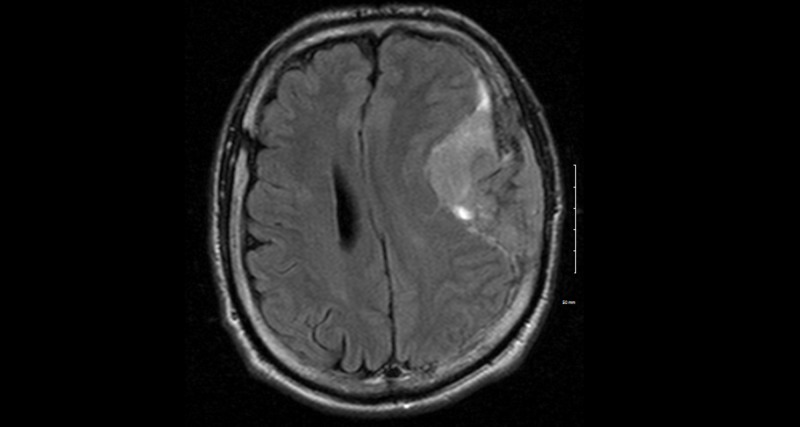
Axial T2 FLAIR post-contrast sequence showing large left frontal/parietal extra-axial enhancing lesion FLAIR: fluid-attenuated inversion recovery

A previously healthy, 52-year-old Caucasian man presented to his family physician a week after having a tonic-clonic seizure. A magnetic resonance imaging (MRI) scan showed a 10 cm left frontal tumor, which was confirmed as an atypical meningioma following craniotomy and resection (Figure 1).

Postoperatively, he took 400 mg of phenytoin PO once a day. He had no seizures postoperatively or afterward. The patient uneventfully received 60 Gy of adjuvant radiation therapy to the postoperative bed in 30 fractions. Three months  after the resection of the tumor, the patient began a trial of phenytoin but nine days later, he developed symptoms consistent with a generalized seizure. He resumed his daily phenytoin prophylaxis with good effect.

**Figure 2:**
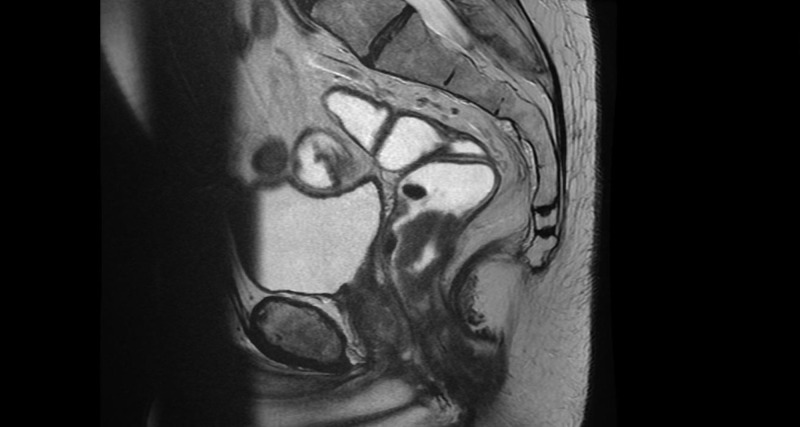
Sagittal T2 image showing low rectal tumor with rectal barium contrast

Two months later, he complained of blood in the stool and after an evaluation was diagnosed with a locally advanced nonmetastatic adenocarcinoma of the low rectum (Figure 2). A curative-intent dose of 50.4 Gy in 28 fractions of  neoadjuvant radiation therapy was prescribed, with 2000 mg PO BID of concurrent radiosensitizing capecitabine [3]. After 20 of the planned 28 fractions, he began to feel unwell and experienced new, right-sided upper and lower limb dysfunction and an unsteady gait. A contrast-enhanced computed tomography (CT) scan of the brain showed no suspicious findings but his phenytoin level was dramatically elevated at 138 µmol/L, compared to 49 µmol/L just prior to neoadjuvant therapy (normal range: 40-80 µmol/L). His albumin level from a few weeks prior to these symptoms had also been normal at 39 g/L (normal range: 34-46 g/L), and he was taking no other medications other than an occasional stimulant laxative. Capecitabine was discontinued, and the patient was treated with charcoal and admitted for observation. Phenytoin was temporarily discontinued and then reintroduced at the previous dose of 400 mg PO per day once levels began to normalize. His symptoms quickly resolved and he showed no further toxicity.

He resumed radiation therapy a few days later without concurrent capecitabine. It was believed that he had developed phenytoin toxicity secondary to impaired clearance as a result of his capecitabine. His phenytoin levels were monitored during the following weeks and his phenytoin dose was bridged with lacosamide and titrated down gradually and then discontinued, with no further symptoms of toxicity. The patient remained on 200 mg PO per day of lacosamide. He underwent a surgical resection with clear margins followed by adjuvant capecitabine and showed no signs of a recurrence of rectal adenocarcinoma thereafter. Three years later, the patient passed away from recurrent meningioma.

## Discussion

Phenytoin’s low aqueous solubility and high degree of plasma protein binding (90%) predispose it to pharmacokinetic interactions, a large number of which have been reported in the literature [1,4]. However, only a small number of reports describe interactions between phenytoin and chemotherapy agents [2]. Case reports have suggested that phenytoin toxicity can result from an interaction with capecitabine [2,5-7]. It is assumed that the interaction is due to the inhibition of the CYP2C9 isoenzyme system by capecitabine [5]. CYP2C9 is a component of the cytochrome p450 group of enzymes and plays a major role in metabolizing phenytoin [2]. Only the free unbound phenytoin has biological activity, so when the reduction in the saturating substrate concentration of phenytoin is reduced, in this case by capecitabine, it causes decreased clearance and an increased serum concentration of phenytoin. Even within the therapeutic range, metabolic mechanisms are saturated and elimination becomes dose-dependent, which explains the narrow therapeutic range of phenytoin [1]. Capecitabine is an oral prodrug, which is metabolized to its active form, 5-fluorouracil (5-FU), by an enzyme found in higher concentrations in solid tumor cells [8]. This localization allows for a more targeted intratumoral release of active 5-FU and, subsequently, less systemic toxicity compared with the intravenous (IV) administration of active 5-FU, but it also means that capecitabine likely has similar effects on the metabolism of phenytoin.

Rosemergy and Findlay reported a case of a phenytoin interaction in a woman receiving adjuvant 5-FU in the treatment of rectal cancer who presented with unsteadiness and blurred vision [5]. They applied the adverse drug reaction probability scale of Naranjo and the interaction was scored as a probable causal relationship. Similarly, Gilbar and Brodribb described a man who had been taking phenytoin for the treatment of epilepsy for over four years, who suddenly became unsteady on his feet after commencing 5-FU therapy for colon cancer [2]. The phenytoin dose was decreased and monitored until chemotherapy was complete and then the normal dose was reinstated with no further signs of toxicity. Brickell and colleagues described very similar cases of phenytoin toxicity in two patients with colorectal cancer receiving 5-FU and one patient with breast cancer receiving capecitabine [6]. Symptoms and serum phenytoin levels normalized after temporary titration followed by the completion of chemotherapy. Although some of these cases suggest that unknown or variable pre-chemotherapy serum phenytoin levels may have increased the risk of toxicity, it seems unlikely that the toxicities experienced by our patient could be attributed to improper phenytoin dosing. He had been on a stable dose for over six months with normal serum levels; toxicity only occurred after he started taking capecitabine. When his capecitabine was discontinued, maintaining his phenytoin levels was no longer a challenge. Other potential explanations for his toxicities also seem unlikely: he was not suffering from hypoalbuminemia or renal failure, and he was not taking other medications that could have competed for albumin binding sites [2].

These cases highlight the importance of patients undergoing full medication reconciliation prior to commencing chemotherapy. Patients on phenytoin should have their serum levels drawn prior to treatment and be on a stable dosing schedule. Levels should be monitored during and following treatment, especially in response to new neurologic symptoms. Miyazaki and colleagues have published a phenytoin-capecitabine interaction model that can help predict the required phenytoin dose adjustments required to keep levels within the therapeutic range [9].

## Conclusions

We believe our patient’s case convincingly demonstrates the risk of phenytoin toxicity due to capecitabine. We suggest that phenytoin levels should be monitored closely during a concurrent administration of capecitabine to avoid symptoms of toxicity.
